# The Des Moines Toothbrush Lecture*Read before the National Dental Association, Boston, Massachusetts, August 23-27, 1920. Printed in July *Journal N. D. A*.

**Published:** 1921-09

**Authors:** C. M. Kennedy

**Affiliations:** Des Moines, Iowa


### THE DES MOINES TOOTHBRUSH LECTURE*

BY C. M. KENNEDY, D.D.S.. DES MOINES, IOWA

Until the people learn what food to eat, when and how to prepare and masticate it, and control their habits and mode of living to such an extent that they will build their bodies to a state of relative immunity to the many oral and dental lesions so prevalent today in early life, it is the duty of the dentist to abort or prevent the consequences that are constantly obtaining because of gross neglect of our physical well-being.

While certain health authorities are endeavoring to teach the public these essentials, the importance of which was recently emphasized when the Republican party incorporated in its platform the combining of the government education and health activities for the better propagation of the correlating bodies, greater success can be obtained by the government and other similarly interested organizations, which the public will not only welcome but praise, including the efforts of the dental profession collectively and individually, if each practitioner will do his part sincerely and intelligently whether it be from a humanitarian standpoint or otherwise.

Because of the almost negligible amount of preventive dentistry that is really being taught and successfully applied by the general practitioner, the Des Moines Toothbrush Clinic was organized. The purpose was to give to the dental profession a definite effective procedure for the educating of their patients in the elimination of mouth bacteria, the ever-present and potent factors in every case of dental caries and pyorrhea alveolaris—two of the most prevalent diseases to which man is heir—together with the

*Read before the National Dental Association, Boston, Massachusetts, August 23-27, 1920. Printed in July *Journal N. D. A.*

many other oral lesions, which are the predisposing causes of many serious systemic disturbances.

Ever since the dental professions inception, its entire efforts have been exerted in combating these ravages, and today we find they are more prevalent than ever. Even in the mouths of our patients whom we have previously treated and advised (and I fear worthless advice is generally what they received), we find them returning with oral conditions but slightly improved, if at all, regardless of the fact that they have put forth their best efforts to avoid a recurrence of the past conditions. There is no branch or specialty practiced by the dental profession which precludes a general knowledge of oral prophylaxis and as every practitioner considers his patient's health and well-being paramount, he must persistently endeavor to effectively teach preventive dentistry.

In the past, as I have suggested, the effort of the dentist has been too often restricted to a few words of advice about home procedures. Never has he offered to show the patient by taking a toothbrush and demonstrating how to proceed and where the most effective results could be obtained. This is due largely to the fact, I believe, that the practitioner cannot properly brush his own mouth nor has he taught his assistant. Neither can he refer to patients who themselves are keeping their mouths relatively immune by the methods he really has taught them. Many are the praises sung for the more or less temporary repair of damages already done, but how many patients are lauding their dentists for having inspired in them a certain amount of assurance that a recurrence of a lesion in that or some other part of the mouth is lessened in proportion to the amount of preventive effort put forth on their part? Most people are accustomed to dirty teeth and it is unusual when they feel clean. This is just the opposite as it pertains to other parts of the body, and is there harm in calling the laity's attention to this fact?

Where is decay most frequently found? Do the patients know that its beginnings are far more prevalent on the mesial, occusal, and distal surfaces, or, in simpler words for them, between the teeth and on the pitted chewing surfaces? They should make examinations of their own teeth and be made to realize that the unclean surfaces are the ones that decay, instead of being told the old phrase, "A clean tooth never decays," which has become incomprehensible to the average individual. Then that less painful but much dreaded and persistent systemically injurious disease, pyorrhea alveolaris, which so frequently originates in an unclean area at the gingivae, recovers most wonderfully by a maintained cleanliness of those areas. Many other species of bacteria, such as the tuberculosis, pneumonia, diphtheria and other bacilli that are constantly present or continually developing in the mouth, where they have a most favorable environment of heat, moisture and food, can with proper toothbrushing and rinsing of the mouth be removed to such an extent that a state of cleanliness will be maintained which will greatly increase one's immunity to many contagious diseases, the greater percentage of which enter the body through the oral cavity.

A number of average cases can be shown where bacteria counts have been made before instructing the patient in the care of the mouth and a count made thereafter for three consecutive weeks, and in every case there is a reduction of more than 90 per cent. This is a test that any practitioner can make wherever a competent pathologist is available, and it is a very convincing argument for proper brushing. Cases of rheumatism, malaise and mental diseases have been greatly relieved by correct toothbrushing and mouth rinsing. Other equally interesting demonstrations can be made, much to the satisfaction of the dentist and intensely pleasing to the patient, because of the possible avoidance of a recurrence of the disease due to an intelligent effort on his part.

While proper brushing is effective as a cure, its greatest merits are in real prevention. Thus the public will have more respect and confidence in the dentist when he can teach them that most all tooth surfaces can be cleansed with a toothbrush and that most of the brushing being done nowadays only hits the high spots, where decay seldom starts. Attention should be called to the fact that there are five surfaces on every tooth to be brushed, mesial, occlusal, distal, buccal, and lingual. The dentist should use a molar tooth for an example and for convenience substitute the words, front, top, back outside and inside in the order that I have named them. Brushing outside means only one-fifth of a job, brushing outside and inside means two fifths of a job, and that is where most people stop, thinking they have met the requirements, when in reality these two surfaces probably would be the least affected by decay if no toothbrushes were used. Proper brushing of the front, top and back sides (mesial, occlusal and distal surfaces) should be insisted upon and the other two surfaces will unavoidably be well brushed. The brush should be placed at right angles to the long axis of the teeth with the points of the bristles in contact with the outer surfaces, and the back of the brush inclined but slightly toward the gums; then the bristles should be gently forced in between the teeth and the brush given several slight rotary or vibratory movements, so that the sides of the bristles come in contact with the gum tissues, care being exercised not to remove the bristles from between the teeth. After a sufficient number of applications have been made in an area, the brush should be removed entirely and carried to the next area; the bristles should not be dragged from one location to another, for they will not properly enter the next embrasures but will either double up or glance off the tooth surface and strike the gums at an angle that will be painful or injurious. This operation should be continued in regular successive steps, every embrasure being entered from both the outer and inner surfaces on both the upper and lower teeth. Some third molars, because of the close proximity of the ramus of the jaw and the soft tissues in that region, cannot be approached at right angles; therefore the brush can be tipped so that the bristle ends reach the out and back sides (buccal and distal surfaces). Third molars in proper alignment and occlusion serve as long as any other teeth if they are kept equally clean. Where a tooth is missing, it is necessary to slightly change the angle of the brush so that the bristle ends come in contact with the back side (distal surface) of the forward tooth and the front side (mesial surface) of the back tooth. Lastly but equally important, the chewing or occlusal surfaces should not be brushed with a sliding or sweeping motion, but the brush placed directly upon this surface and the bristles gently forced into the pits and fissures and then given a slight rotary movement. It should be removed and repeated in each successive area until the entire chewing surface has been brushed. While this entire procedure seems long in the telling, in reality, after a little thoughtful practice, it will require no longer time than the patients generally claim they are expending by their own methods.

After this has been completed, what has been accomplished? No appreciable amount of debris or number of bacteria has been removed from the mouth as yet. The accumulations have been only loosened from their abode. Let us consider their removal from the mouth by proceeding to thoroughly rinse every corner and crevice. Water flows in the direction of the least resistance and when you take water into the mouth and force it over the entire area with the lips closed, but jaws apart, you are again flushing only the surfaces that need the least attention. The jaws should be held in firm occlusion and the water vigorously forced through the embrasures in a definite location, fresh water being used for each section of the mouth.

Good habits are best established early in life and this law may be applied beneficially in the care of the deciduous teeth, which are of great importance to the erupting permanent teeth, for a healthy individual cannot enter an infected house without greatly increasing his ability to succumb to the infection.

Furthermore, all sincere competent practitioners take great pride in the restorations and remedies they make in the mouth, but they realize that their best is not to be compared with what nature has done and can do when she is not interfered with. Therefore they should impress upon the patient's mind that in most cases he was first provided with the best known dental armory but that in its past environment parts of it have failed, due to mode of living, or to lack of care, or to both, and unless that condition is greatly improved he cannot hope or expect the new restorations to compare with the orginals in either efficiency or endurance. Acceptance of this information by patients and care of the substitutes will have a twofold effect; it will please and inspire the practitioner and it will give better service and satisfaction to the patients.

I would urge every practitioner to give mouth cleanliness more consideration. The public is becoming more interested every day, as is manifested by the crowded attendance at the public and municipal clinics already organized and by the number of clinics that are now being started with the co-operation of the public. Many large industries are establishing dental clinics and consider it a profitable investment. The modern dentist is not selling fillings, bridges and plates but is selling health and the conscientious dentist will put forth every effort to acquaint himself with every development in preventive dentistry so that he may help to keep his patients in the enjoyment of good health, and prolong their years of usefulness.

